# 1,1′-(Propane-1,3-di­yl)bis­(3-phenyl­urea)

**DOI:** 10.1107/S1600536811035343

**Published:** 2011-09-03

**Authors:** Pramod Pansuriya, Hariska Naidu, Holger B. Friedrich, Glenn E. M. Maguire

**Affiliations:** a School of Chemistry, University of KwaZulu–Natal, Durban 4000, South Africa

## Abstract

The title compound, C_17_H_20_N_4_O_2_, has crystallographic inversion symmetry. In the crystal structure, inter­molecular hydrogen bonding between adjacent urea groups gives rise to infinite polymeric chains diagonally across the *bc* plane. With a centroid–centroid distance of 3.295 (2) Å, π–π stacking is present in the crystal along the same plane.

## Related literature

For applications of ureas, see: Park *et al.* (2011 ▶); Ahmed *et al.* (2011 ▶); Sharma *et al.* (2010 ▶); Vos *et al.* (2010 ▶); Dawn *et al.* (2011 ▶). For related structures, see: Koevoets *et al.* (2005 ▶).
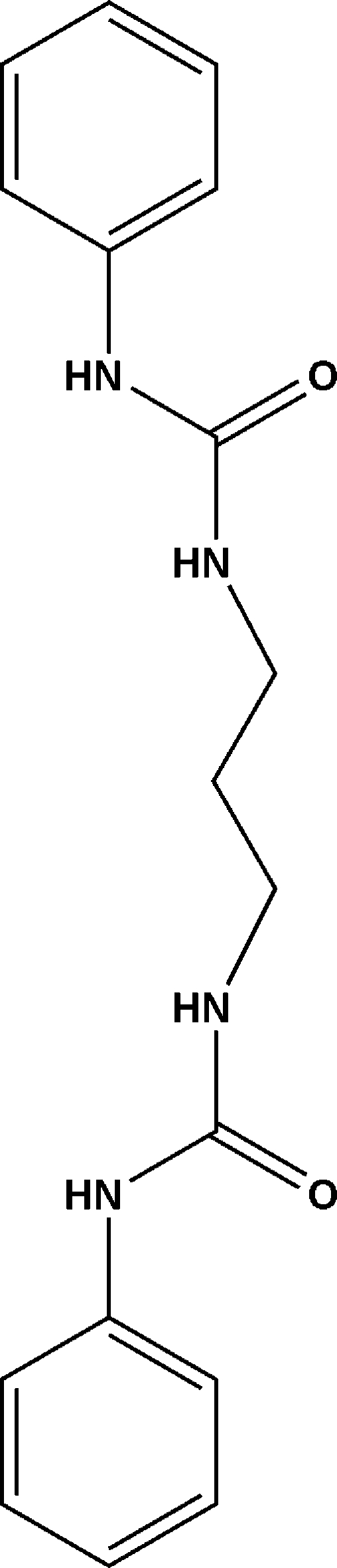


## Experimental

### 

#### Crystal data

C_17_H_20_N_4_O_2_
*M* *_r_* = 312.37
Monoclinic, 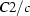

*a* = 33.811 (7) Å
*b* = 4.598 (1) Å
*c* = 9.891 (2) Å
β = 98.957 (4)°
*V* = 1518.9 (6) Å^3^
*Z* = 4
Mo *K*α radiation
μ = 0.09 mm^−1^
*T* = 173 K
0.50 × 0.21 × 0.02 mm

#### Data collection

Bruker Kappa DUO APEXII diffractometer
Absorption correction: multi-scan (*TWINABS*; Sheldrick, 2007 ▶)*T*_min_ = 0.955, *T*_max_ = 0.998
1930 measured reflections
1930 independent reflections
1811 reflections with *I* > 2σ(*I*)
*R*_int_ = 0.042

#### Refinement

*R*[*F* ^2^ > 2σ(*F* ^2^)] = 0.033
*wR*(*F* ^2^) = 0.092
*S* = 1.05
1930 reflections
114 parameters
H atoms treated by a mixture of independent and constrained refinement
Δρ_max_ = 0.27 e Å^−3^
Δρ_min_ = −0.19 e Å^−3^

### 

Data collection: *APEX2* (Bruker, 2006 ▶); cell refinement: *SAINT* (Bruker, 2006 ▶); data reduction: *SAINT*; program(s) used to solve structure: *SHELXS97* (Sheldrick, 2008 ▶); program(s) used to refine structure: *SHELXL97* (Sheldrick, 2008 ▶); molecular graphics: *OLEX2* (Dolomanov *et al.*, 2009 ▶); software used to prepare material for publication: *SHELXL97*.

## Supplementary Material

Crystal structure: contains datablock(s) I, global. DOI: 10.1107/S1600536811035343/hg5067sup1.cif

Structure factors: contains datablock(s) I. DOI: 10.1107/S1600536811035343/hg5067Isup2.hkl

Supplementary material file. DOI: 10.1107/S1600536811035343/hg5067Isup3.cml

Additional supplementary materials:  crystallographic information; 3D view; checkCIF report

## Figures and Tables

**Table 1:** Hydrogen-bond geometry (Å, °)

*D*—H⋯*A*	*D*—H	H⋯*A*	*D*⋯*A*	*D*—H⋯*A*
N1—H1*N*⋯O1^i^	0.834 (18)	2.124 (18)	2.8742 (14)	149.7 (13)
N2—H2*N*⋯O1^i^	0.864 (18)	2.119 (18)	2.8904 (14)	148.4 (15)

Symmetry code: (i) 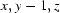.

**Fig. 1.**
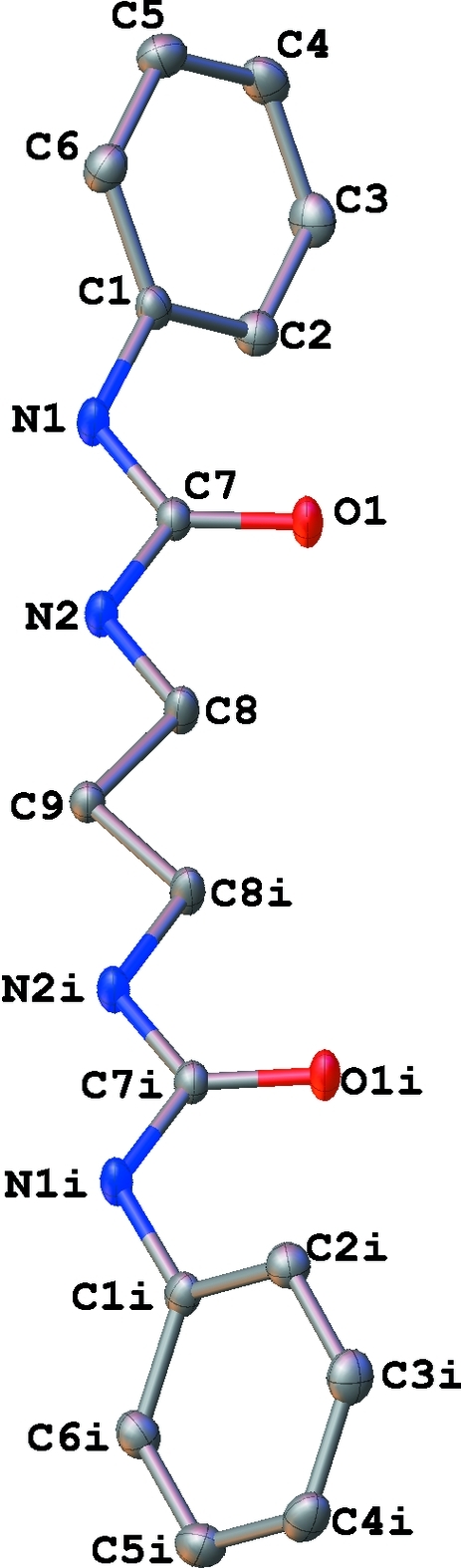
The molecular structure of the title compound with atomic numbering scheme. The hydrogen atoms have been omitted clarity. Displacement elipsoids are drawn at 40% probability. The symmetry code is (i) : 1-x,y, 1.5-z.

**Fig. 2.**
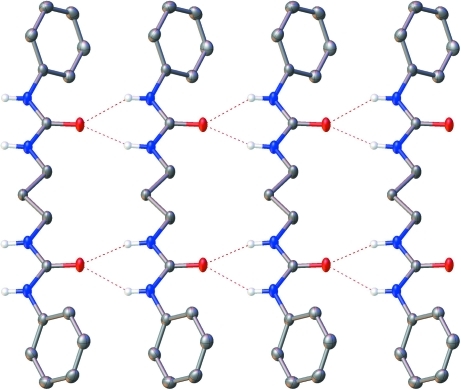
The hydrogen bonding interactions of the title compound along the [001] axis. All hydrogen atoms except those involved in hydrogen bonding interactions have been omitted for clarity. Displacement elipsoids are drawn at 40% probability.

## supplementary crystallographic information

### Comment

Bis-ureas have been employed as ligands for metal complexes used in hydrolytic kinetic resolution of epoxides (Park *et al.*, 2011) and as chromogenic and fluorogenic receptors (Ahmed *et al.*, 2011). These molecules have also been found to be useful as epigenetic modulators (Sharma *et al.*, 2010), in surfactant self-assembies (Vos *et al.*, 2010), and photo dimerizing agent for coumarins (Dawn *et al.*, 2011).

The closest reported structures are 3,3'-bis-phenyl-(butylene-1,4)-bisurea and 3,3'-bis-phenyl-(heptylene-1,7)-bisurea (Koevoets *et al.*, 2005). In the butylene derivatives a *transoid* arrangement is evident whereas the heptylene molecule adopts a *cisoid* arrangement of the two urea groups. The title compound has an odd number of carbons in its aliphatic chain (propylene). This leads to a *cisoid* arrangement of the two urea groups (Fig. 1).

The asymmetric unit of the title compund, C_17_H_20_N_4_O_2_, contains half molecule of 1,1'-(propane-1,3-diyl)bis(3-phenylurea) and the complete molecule is generated by inversion symmetry (i) : 1-*x*, *y*, 1.5-*z*. Intermolecular hydrogen bonding between adjacent urea groups N1–H1–O1, 2.8742 (14) Å and N2–H2–O1, 2.8904 (14) Å gives rise to infinite polymeric chains across the *bc* plane (Fig. 2), The spacing between the two hydrogen-bonded urea groups is 4.59 Å in the title compound, while it is 4.64 Å for the even butylene spacer and 4.63 Å for the odd heptylene spacer.  With a centroid distance of less than 3.5 Å, π-π stacking is present in the crystal along the same plane.

### Experimental

A solution of phenyl isocyanate (6.76 g, 50 mmol) in diethylether (15 ml) was added dropwise at 15 °C to a vigorously stirred solution of anhydrous propane-1,3-diamine (7.41 g, 100 mmol) in isopropyl alcohol (100 ml) over a period of 30 min.The reaction mixture was stirred for 2 hrs at room temperature and quenched with water (200 ml). The reaction mixture was maintained overnight at room temperature. Then the reaction mixture was acidified with conc. HCl to pH 2.6. The solvents were evaporated under vacuum, the residue was suspended in hot water for 30 min and the resulting precipitate was filtered. The product was washed with ice cold water and dried. The yield was 2.70 g (40%).

Crystals suitable for single-crystal X-ray diffraction were grown in methanol: methylenechloride (1:2) at room temperature. *M*.p. = 504 K.

### Refinement

All non-hydrogen atoms were refined anisotropically. All hydrogen atoms, except the H atoms H1N and H2N on N1 and N2, were positioned geometrically with C—H distances ranging from 0.95 Å to 0.99 Å and refined as riding on their parent atoms with *U*_iso_ (H) = 1.2*U*_eq_ (C). The positions of H1N and H2N were located in the difference electron density maps and refined independently.

### Crystal data

**Table:**

C_17_H_20_N_4_O_2_	*F*(000) = 664
*M**_r_* = 312.37	*D*_x_ = 1.366 Mg m^−^^3^
Monoclinic, *C*2/*c*	Melting point: 504 K
Hall symbol: -C 2yc	Mo *K*α radiation, λ = 0.71073 Å
*a* = 33.811 (7) Å	Cell parameters from 1930 reflections
*b* = 4.598 (1) Å	θ = 2.4–28.5°
*c* = 9.891 (2) Å	µ = 0.09 mm^−^^1^
β = 98.957 (4)°	*T* = 173 K
*V* = 1518.9 (6) Å^3^	Plate, colourless
*Z* = 4	0.50 × 0.21 × 0.02 mm

### Data collection

**Table:**

Bruker Kappa DUO APEXII diffractometer	1930 independent reflections
Radiation source: fine-focus sealed tube	1811 reflections with *I* > 2σ(*I*)
graphite	*R*_int_ = 0.042
0.5° φ scans and ω scans	θ_max_ = 28.5°, θ_min_ = 2.4°
Absorption correction: multi-scan (TWINABS; Sheldrick, 2007)	*h* = −44→44
*T*_min_ = 0.955, *T*_max_ = 0.998	*k* = 0→6
1930 measured reflections	*l* = 0→13

### Refinement

**Table:**

Refinement on *F*^2^	Primary atom site location: structure-invariant direct methods
Least-squares matrix: full	Secondary atom site location: difference Fourier map
*R*[*F*^2^ > 2σ(*F*^2^)] = 0.033	Hydrogen site location: inferred from neighbouring sites
*wR*(*F*^2^) = 0.092	H atoms treated by a mixture of independent and constrained refinement
*S* = 1.05	*w* = 1/[σ^2^(*F*_o_^2^) + (0.0565*P*)^2^ + 0.4485*P*] where *P* = (*F*_o_^2^ + 2*F*_c_^2^)/3
1930 reflections	(Δ/σ)_max_ = 0.001
114 parameters	Δρ_max_ = 0.27 e Å^−^^3^
0 restraints	Δρ_min_ = −0.19 e Å^−^^3^

### Special details

**Table:**

Geometry. All e.s.d.'s (except the e.s.d. in the dihedral angle between two l.s. planes) are estimated using the full covariance matrix. The cell e.s.d.'s are taken into account individually in the estimation of e.s.d.'s in distances, angles and torsion angles; correlations between e.s.d.'s in cell parameters are only used when they are defined by crystal symmetry. An approximate (isotropic) treatment of cell e.s.d.'s is used for estimating e.s.d.'s involving l.s. planes.
Refinement. Refinement of *F*^2^ against ALL reflections. The weighted *R*-factor *wR* and goodness of fit *S* are based on *F*^2^, conventional *R*-factors *R* are based on *F*, with *F* set to zero for negative *F*^2^. The threshold expression of *F*^2^ > σ(*F*^2^) is used only for calculating *R*-factors(gt) *etc*. and is not relevant to the choice of reflections for refinement. *R*-factors based on *F*^2^ are statistically about twice as large as those based on *F*, and *R*- factors based on ALL data will be even larger.

### Fractional atomic coordinates and isotropic or equivalent isotropic displacement parameters (Å^2^)

**Table:**

	*x*	*y*	*z*	*U*_iso_*/*U*_eq_	Occ. (<1)
O1	0.42150 (2)	0.94129 (16)	0.45600 (10)	0.0258 (2)	
N1	0.39232 (3)	0.5132 (2)	0.37683 (12)	0.0243 (2)	
H1N	0.3915 (4)	0.335 (4)	0.3917 (18)	0.035 (4)*	
N2	0.44667 (3)	0.5183 (2)	0.54828 (11)	0.0228 (2)	
H2N	0.4448 (5)	0.331 (4)	0.5513 (17)	0.039 (5)*	
C1	0.36029 (3)	0.6418 (2)	0.28699 (11)	0.0204 (2)	
C2	0.36739 (3)	0.8527 (3)	0.19350 (13)	0.0243 (2)	
H2	0.3940	0.9150	0.1897	0.029*	
C3	0.33567 (4)	0.9726 (3)	0.10557 (14)	0.0285 (3)	
H3	0.3405	1.1188	0.0423	0.034*	
C4	0.29688 (4)	0.8801 (3)	0.10954 (14)	0.0297 (3)	
H4	0.2752	0.9631	0.0494	0.036*	
C5	0.28992 (4)	0.6679 (3)	0.20069 (14)	0.0303 (3)	
H5	0.2633	0.6025	0.2023	0.036*	
C6	0.32140 (4)	0.5478 (3)	0.29065 (14)	0.0267 (3)	
H6	0.3164	0.4026	0.3541	0.032*	
C7	0.42011 (3)	0.6716 (2)	0.46010 (12)	0.0194 (2)	
C8	0.47434 (3)	0.6748 (2)	0.64875 (13)	0.0247 (3)	
H8A	0.4590	0.8079	0.6998	0.030*	
H8B	0.4921	0.7951	0.6007	0.030*	
C9	0.5000	0.4779 (3)	0.7500	0.0195 (3)	
H9B	0.4829	0.3523	0.7980	0.023*	0.50
H9A	0.5171	0.3523	0.7020	0.023*	0.50

### Atomic displacement parameters (Å^2^)

**Table:**

	*U*^11^	*U*^22^	*U*^33^	*U*^12^	*U*^13^	*U*^23^
O1	0.0313 (4)	0.0117 (4)	0.0306 (4)	0.0004 (3)	−0.0070 (4)	−0.0002 (3)
N1	0.0283 (4)	0.0126 (4)	0.0281 (5)	−0.0010 (4)	−0.0078 (4)	0.0011 (4)
N2	0.0262 (4)	0.0130 (4)	0.0260 (5)	−0.0007 (3)	−0.0057 (4)	0.0003 (4)
C1	0.0240 (5)	0.0158 (5)	0.0195 (5)	0.0011 (4)	−0.0025 (4)	−0.0027 (4)
C2	0.0259 (5)	0.0235 (5)	0.0228 (6)	−0.0003 (4)	0.0018 (4)	0.0003 (5)
C3	0.0366 (6)	0.0261 (6)	0.0215 (5)	0.0016 (5)	0.0008 (5)	0.0045 (5)
C4	0.0295 (6)	0.0282 (6)	0.0277 (6)	0.0056 (5)	−0.0073 (5)	−0.0024 (5)
C5	0.0239 (5)	0.0311 (6)	0.0343 (7)	−0.0022 (4)	−0.0010 (5)	−0.0025 (5)
C6	0.0291 (5)	0.0237 (5)	0.0256 (6)	−0.0042 (4)	−0.0005 (5)	0.0014 (5)
C7	0.0225 (5)	0.0149 (4)	0.0201 (5)	0.0004 (4)	0.0006 (4)	−0.0007 (4)
C8	0.0273 (5)	0.0148 (5)	0.0280 (6)	−0.0005 (4)	−0.0086 (5)	−0.0002 (4)
C9	0.0205 (6)	0.0147 (6)	0.0216 (7)	0.000	−0.0023 (6)	0.000

### Geometric parameters (Å, °)

**Table:**

O1—C7	1.2416 (13)	C3—H3	0.9500
N1—C7	1.3607 (14)	C4—C5	1.373 (2)
N1—C1	1.4187 (14)	C4—H4	0.9500
N1—H1N	0.833 (19)	C5—C6	1.3909 (17)
N2—C7	1.3492 (14)	C5—H5	0.9500
N2—C8	1.4463 (14)	C6—H6	0.9500
N2—H2N	0.862 (19)	C8—C9	1.5180 (14)
C1—C2	1.3866 (17)	C8—H8A	0.9900
C1—C6	1.3898 (17)	C8—H8B	0.9900
C2—C3	1.3857 (16)	C9—C8^i^	1.5180 (14)
C2—H2	0.9500	C9—H9B	0.9900
C3—C4	1.3850 (19)	C9—H9A	0.9900
			
C7—N1—C1	122.95 (9)	C6—C5—H5	119.7
C7—N1—H1N	117.3 (11)	C1—C6—C5	119.51 (13)
C1—N1—H1N	118.5 (11)	C1—C6—H6	120.2
C7—N2—C8	118.57 (9)	C5—C6—H6	120.2
C7—N2—H2N	119.8 (11)	O1—C7—N2	121.22 (10)
C8—N2—H2N	121.1 (11)	O1—C7—N1	122.76 (10)
C2—C1—C6	119.85 (11)	N2—C7—N1	116.02 (9)
C2—C1—N1	120.98 (11)	N2—C8—C9	113.49 (9)
C6—C1—N1	119.15 (11)	N2—C8—H8A	108.9
C3—C2—C1	119.95 (11)	C9—C8—H8A	108.9
C3—C2—H2	120.0	N2—C8—H8B	108.9
C1—C2—H2	120.0	C9—C8—H8B	108.9
C4—C3—C2	120.25 (13)	H8A—C8—H8B	107.7
C4—C3—H3	119.9	C8^i^—C9—C8	106.78 (12)
C2—C3—H3	119.9	C8^i^—C9—H9B	110.4
C5—C4—C3	119.78 (11)	C8—C9—H9B	110.4
C5—C4—H4	120.1	C8^i^—C9—H9A	110.4
C3—C4—H4	120.1	C8—C9—H9A	110.4
C4—C5—C6	120.65 (12)	H9B—C9—H9A	108.6
C4—C5—H5	119.7		
			
C7—N1—C1—C2	53.70 (18)	N1—C1—C6—C5	−178.50 (11)
C7—N1—C1—C6	−128.16 (14)	C4—C5—C6—C1	−0.7 (2)
C6—C1—C2—C3	1.05 (18)	C8—N2—C7—O1	6.54 (18)
N1—C1—C2—C3	179.18 (11)	C8—N2—C7—N1	−173.82 (12)
C1—C2—C3—C4	−0.77 (19)	C1—N1—C7—O1	−6.0 (2)
C2—C3—C4—C5	−0.2 (2)	C1—N1—C7—N2	174.39 (12)
C3—C4—C5—C6	1.0 (2)	C7—N2—C8—C9	174.67 (10)
C2—C1—C6—C5	−0.34 (19)	N2—C8—C9—C8^i^	−177.37 (13)

Symmetry codes: (i) −*x*+1, *y*, −*z*+3/2.

### Hydrogen-bond geometry (Å, °)

**Table:**

*D*—H···*A*	*D*—H	H···*A*	*D*···*A*	*D*—H···*A*
N1—H1N···O1^ii^	0.834 (18)	2.124 (18)	2.8742 (14)	149.7 (13)
N2—H2N···O1^ii^	0.864 (18)	2.119 (18)	2.8904 (14)	148.4 (15)

Symmetry codes: (ii) *x*, *y*−1, *z*.
